# An attention-augmented multimodal classification of alzheimer’s disease and parkinson’s disease vs healthy controls using MRI, EEG, and SNP data

**DOI:** 10.1038/s41598-025-32274-6

**Published:** 2026-01-16

**Authors:** Basu Dev Shivahare, Hariharan Rajadurai, Deeba K, Sahil Kansal, Sandeep Kumar Mathivanan, Sangeetha S.K.B

**Affiliations:** 1 School of Computing Science and Engineering, Galgotias University, Greater Noida, 203201 India; 2 School of Computing Science and Engineering, VIT Bhopal University, Bhopal–Indore Highway Kothrikalan, Sehore, 466114 India; 3 School of Computer Science and Applications, REVA University, Bangalore, India; 4 School of Computer Science and Engineering, IILM University, Greater Noida, India; 5 Manipal Institute of Technology Bengaluru, Manipal Academy of Higher Education, Manipal, India

**Keywords:** Multimodal deep learning, Cross-modality attention, Alzheimer’s disease, Parkinson’s disease, MRI, EEG, SNP, Uncertainty estimation, Neurodegenerative disease diagnosis, Computational biology and bioinformatics, Diseases, Neurology, Neuroscience

## Abstract

Due to the late manifestation of structural symptoms and symptomatic overlap, neurodegenerative diseases such as Parkinson’s Disease (PD) and Alzheimer’s Disease (AD) remain difficult to diagnose accurately. In order to categorize AD and PD in comparison to Healthy Controls (HC), this study suggests a multimodal classification framework that combines genetic Single Nucleotide Polymorphism (SNP) data, structural Magnetic Resonance Imaging (MRI), and functional Electroencephalography (EEG). To improve the model’s accuracy and interpretability, the method makes use of an uncertainty estimate module and a novel cross-modality attention mechanism. The framework strives for diagnosis, concentrating on detecting Parkinson’s disease (PD) and Alzheimer’s disease (AD) in individuals exhibiting modest motor symptoms or early cognitive impairments, which are indicative of the prodromal stage of both conditions. A dataset of 2,500 MRI images, 1,500 EEG recordings, and SNP data for 1,000 subjects drawn from OpenNeuro, PPMI, and the UK Biobank was utilized in extensive analyses. The developed model was contrasted with recent unimodal and multimodal techniques. Our findings exhibit statistically significant increases of 6–12% compared to similar methods, with 95.6% average classification accuracy on AD and 94.8% on PD. The importance of the attention mechanism and both modalities to overall performance is quantified using ablation studies. Quantification of uncertainty also improves interpretability for possible clinical use. These results demonstrate the proper neurodegenerative disease diagnosis when explainable AI elements are paired with stable multimodal fusion.

## Introduction

The slow and late manifestation of the structural changes in the brain, and overlap between symptom onset, neurodegenerative conditions like Parkinson’s Disease (PD) and Alzheimer’s Disease (AD) are the most difficult to diagnose at an early stage^1–3^. Conventional diagnostic methods based on clinical symptoms and genetic testing, structural MRI, or functional EEG, even at times the correct or late diagnosis^4,5^. While each approach has some potential as a therapy, both are narrow in application and generally will detect only disease markers after enormous quantities of neuronal loss have taken place^6,7^.

Structural MRI has shown potential for identifying anatomical atrophy with advancing sophisticated neuroimaging and molecular methods, most prominently the hippocampus in AD and motor tracts^8,9^. While genetic biomarkers like APOE-ε4 and LRRK2 offer predictive data on illness risk, functional EEG recordings uncover aberrant brain dynamics linking to cognitive or motor impairment^10–13^. These modalities alone are still not sufficient for accurate early-stage detection: MRI is unable to detect preclinical or functional alterations, genetic testing is not yet available and is expensive, and EEG has poor spatial resolution^14,15^.

Multimodal fusion, which combines complementary structural, functional, and genetic data to provide a richer characterization of illness phenotypes, has shown promise in recent publications^16,17^. Despite these advances, current strategies tend to rely on naive fusion techniques, are difficult to interpret, or do not sufficiently leverage important intermodality dependencies. Furthermore, uncertainty quantification, central to clinical acceptability and decision support is not yet prevalent in systems^18^. The proposed study puts forward an Attention-Augmented Multimodal Deep Learning Framework (AAMDLF) that combines MRI, EEG, and SNP data to classify early AD and PD with precision in trying to overcome these constraints.

A new cross-modality attention fusion module is introduced in the framework that learns to rank dynamically the features of every modality according to how relevant they are to a given patient. To enable increased interpretability and trustworthiness, an uncertainty estimation module is also incorporated. Large-scale experiments on large, well-curated UK Biobank, PPMI, and ADNI datasets prove substantial gains over current multimodal and unimodal baselines^19–22^. A correct and personalized benchmark for neurodegenerative disease diagnosis is established with the fusion of multiple data streams based on explainable fusion techniques.

Transformer models have garnered significant attention recently owing to their very effective performance in medical imaging applications as a result of their ability to learn long-range dependencies in data. With the utilization of self-attention mechanisms to represent complex spatial interactions within images, Vision Transformers (ViTs) bested traditional Convolutional Neural Networks (CNNs) in image classification benchmarks through state-of-the-art performance. Transformers have been applied to applications in medical imaging in various studies, including:^23^ proposed a transformer-based framework for self-driving medical picture segmentation, which was more accurate and robust compared to CNN-based methods.

^24^ Enhanced generalization on test data by developing a hybrid model for medical image classification that integrates CNNs with transformers^25^.Utilized transformers for multimodal integration in medical image processing, integrating data from multiple sources, such as MRI and PET scans, to facilitate more accurate disease diagnosis. The emphasis of our research on combining numerous data modalities for enhanced illness identification is consistent with these recent transformer-based advancements, which exhibit their promise to enhance medical imaging models, especially in complex, multimodal tasks.

To enhance the diagnosis of neurodegenerative diseases, more recent studies have focused on multimodal data fusion methods^26^. Introduced SMART, a self-weighted multimodal fusion method that integrates multiple data modalities to enhance the accuracy of neurodegenerative disease diagnosis. Conducting a scoping study using clinically accessible tests^27^, highlighted the growing practice of fusing multiple sources of data to enable more accurate diagnosis^28^. Discussed optimization techniques for the diagnosis of Parkinson’s disease that employ multimodal fusion in order to enhance the effectiveness and accuracy of disease detection^29^. Proposed a deep network using multimodal fusion in order to enhance detection rates so as to effectively diagnose Alzheimer’s disease.

Multimodal gait recognition for neurodegenerative disorders were also studied in^30^, illustrating how the fusion of sensor streams of data could facilitate early disease detection. These works illustrate the promising progresses in multimodal fusion towards more reliable and accurate diagnoses of neurodegenerative diseases^31^. Presents a technique for improving information flow and addressing modality ambiguity to produce more precise longitudinal forecasts^32^. Improves this even more by handling missing data with adaptive cross-modal representation learning, which increases the robustness of illness progression models^33^. Uses latent representation learning to address the problem of insufficient neuroimaging and genetic data, showing promise for precise Alzheimer’s diagnosis in spite of data constraints.

The classification of Alzheimer’s Disease (AD) and Parkinson’s Disease (PD) from Healthy Controls (HC) is challenging due to overlapping symptoms and subtle early-stage changes. This study introduces a multimodal classification framework leveraging MRI, EEG, and SNP data, which allows for accurate differentiation between AD, PD, and HC, offering improved diagnostic precision. To measure the model’s confidence in its predictions, we provide an uncertainty estimation module in this study. This module approximates the posterior distribution of the model’s predictions using Monte Carlo Dropout. The uncertainty is expressed as a combination of aleatoric uncertainty (caused by noise or variability in the data itself) and epistemic uncertainty (caused by ignorance of the data or model limitations). This uncertainty is essential for comprehending how reliable the model’s output is, particularly in situations involving clinical decision-making. It is crucial to remember that this uncertainty relates to prediction confidence and has nothing to do with whether modalities are present in the input data or not.

The main objectives of the study are

1. To design and implement an advanced multimodal deep learning architecture that systematically integrates structural MRI, functional EEG, and genetic SNP data to enable robust detection and differentiation of Alzheimer’s Disease (AD) and Parkinson’s Disease (PD).
2. To develop a novel cross-modality attention fusion mechanism that dynamically learns the relative importance of each data modality for each patient, optimizing feature selection and enhancing classification accuracy under varying symptom patterns.
3. To incorporate an uncertainty estimation module within the framework that quantifies prediction confidence, providing reliable, explainable outputs to support clinical decision-making and strengthen trust in AI-assisted diagnostics.
4. To validate the proposed framework through extensive comparative experiments, including state-of-the-art unimodal and multimodal baselines, detailed ablation studies, and statistical significance testing, using large-scale, real-world datasets (OpenNeuro, PPMI, UK Biobank).

Along with this introduction, Section "Attention- augmented multimodal deep learning (aamdlf) framework" discusses the system methodology, Section "Aamdlf algorithm" depicts the experimental results and discussions followed by conclusion in Section "Conclusion".

## Attention- augmented multimodal deep learning (aamdlf) framework

**Fig. 1:**
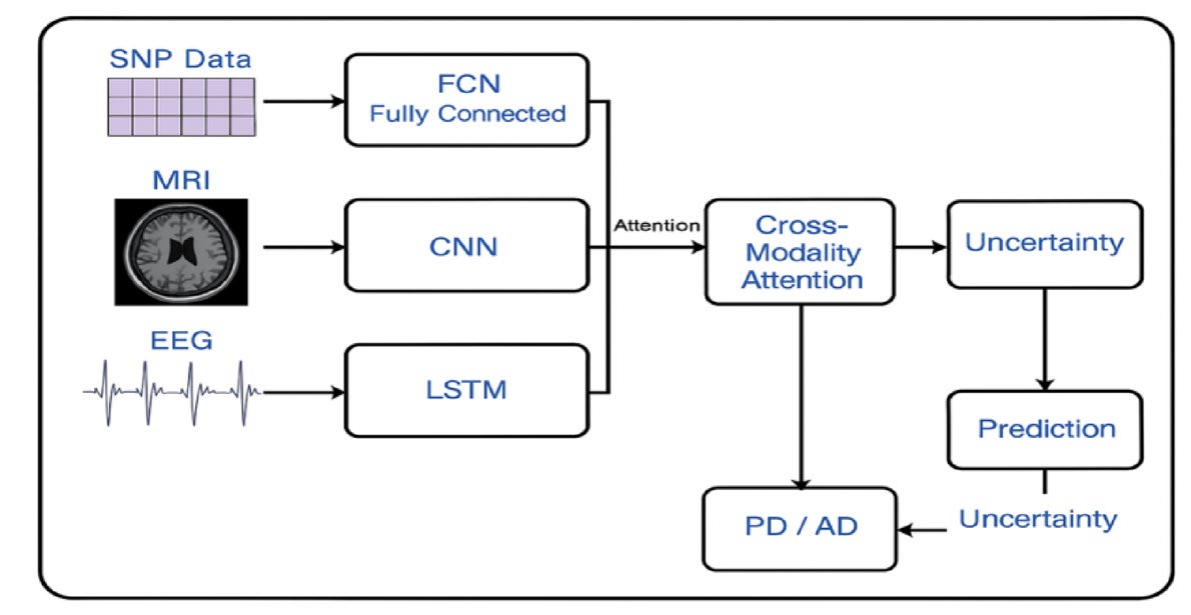
Overview of the Attention-Augmented Multimodal Deep Learning Framework.

The architecture integrates CNNs, LSTMs, and attention mechanisms to extract deep features from MRI and EEG data, process sequential dependencies in SNP data, and apply attention-based fusion across modalities for improved detection accuracy. In Fig. 1, an Attention-Augmented Multimodal Deep Learning Framework (AAMDLF) that simultaneously processes three distinct biomedical data modalities: genetic Single Nucleotide Polymorphism (SNP) data, structural Magnetic Resonance Imaging (MRI) volumes, and functional Electroencephalography (EEG) signals. The input SNP matrix $S\in {R}^{n\times p}$ contains $n$ subjects and $p$ genetic features. The MRI data is defined as a volumetric tensor $M\in {R}^{n\times h\times w\times d}$ where each scan has spatial dimensions height ($h$), width ($w$), and depth ($d$). EEG recordings are represented by $E\in {R}^{n\times T\times c}$, where $T$ is the number of time steps and $c$ is the number of EEG channels.

To ensure robust learning, each SNP feature column is normalized using min–max scaling

1 $${S}_{ij}{\prime}=\frac{{S}_{ij}-min({S}_{j})}{max({S}_{j})-min({S}_{j})}, j\in [1,p]$$

Structural MRI scans undergo N4 bias correction to reduce intensity inhomogeneity. EEG signals are preprocessed using a 0.5–40 Hz bandpass filter and cleaned via Independent Component Analysis (ICA) to eliminate physiological and motion artifacts. For detailed local representation, the 3D MRI volumes are divided into smaller overlapping or non-overlapping patches ${P}_{k}\in {R}^{{p}_{h}\times {p}_{w}\times {p}_{d}}$ with predefined patch dimensions.

The normalized SNP matrix ${S}{\prime}$ is then embedded into a dense representation using a linear layer

2 $${Z}_{S}={W}_{S}{S}{\prime}+{b}_{S}, {W}_{S}\in {R}^{m\times p}, {b}_{S}\in {R}^{m}.$$

MRI volumes are fed into a 3D Convolutional Neural Network (3D-CNN) to extract deep structural features ${F}_{M}={f}_{3D-CNN}(M)$, and EEG signals are passed through a 1D-CNN to capture temporal dynamics ${F}_{E}={f}_{1D-CNN}(E)$.To model sequential dependencies in the SNP data, the embedding ${Z}_{S}$ is further processed by a bidirectional Long Short-Term Memory (Bi-LSTM) network, producing a sequence-aware output ${H}_{S}=BiLSTM({Z}_{S})$. The resulting MRI and EEG features are then flattened into 1D vectors ${F}_{M}{\prime}$ and ${F}_{E}{\prime}$ to standardize dimensionality for the attention modules.

To capture intra-modality dependencies, self-attention mechanisms are applied independently to each modality. For SNP data, query, key, and value matrices are derived

3 $${Q}_{S}={Z}_{S}{W}_{S}^{Q}, {K}_{S}={Z}_{S}{W}_{S}^{K}, {V}_{S}={Z}_{S}{W}_{S}^{V},$$

leading to self-attention weights

4 $${A}_{S}=softmax(\frac{{Q}_{S}{K}_{S}^{T}}{\sqrt{{d}_{k}}}),$$

5 $${O}_{S}={A}_{S}{V}_{S}.$$

Equivalent self-attention computations are performed for the MRI and EEG streams, yielding ${O}_{M}$ and ${O}_{E}$.

**Fig. 2:**
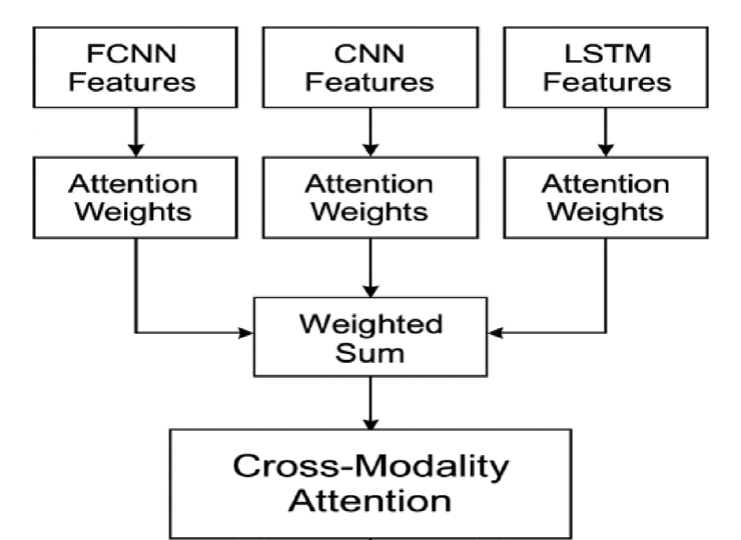
Proposed cross-modality attention.

To exploit inter-modality relationships, the framework includes a cross-modality attention block. For example, SNP features attend to MRI features via

6 $${C}_{SM}=softmax(\frac{{Q}_{S}{K}_{M}^{T}}{\sqrt{{d}_{k}}}){V}_{M},$$

and SNP features also attend to EEG features ${C}_{SE}$. As shown in Fig. 2, MRI features attend to EEG signals ${C}_{ME}$. The self-attended and cross-attended outputs are combined for each modality

7 $${F}_{S}={O}_{S}+{C}_{SM}+{C}_{SE},$$

8 $${F}_{M}{\prime}={O}_{M}+{C}_{SM}+{C}_{ME},$$

9 $${F}_{E}{\prime}={O}_{E}+{C}_{SE}+{C}_{ME}.$$

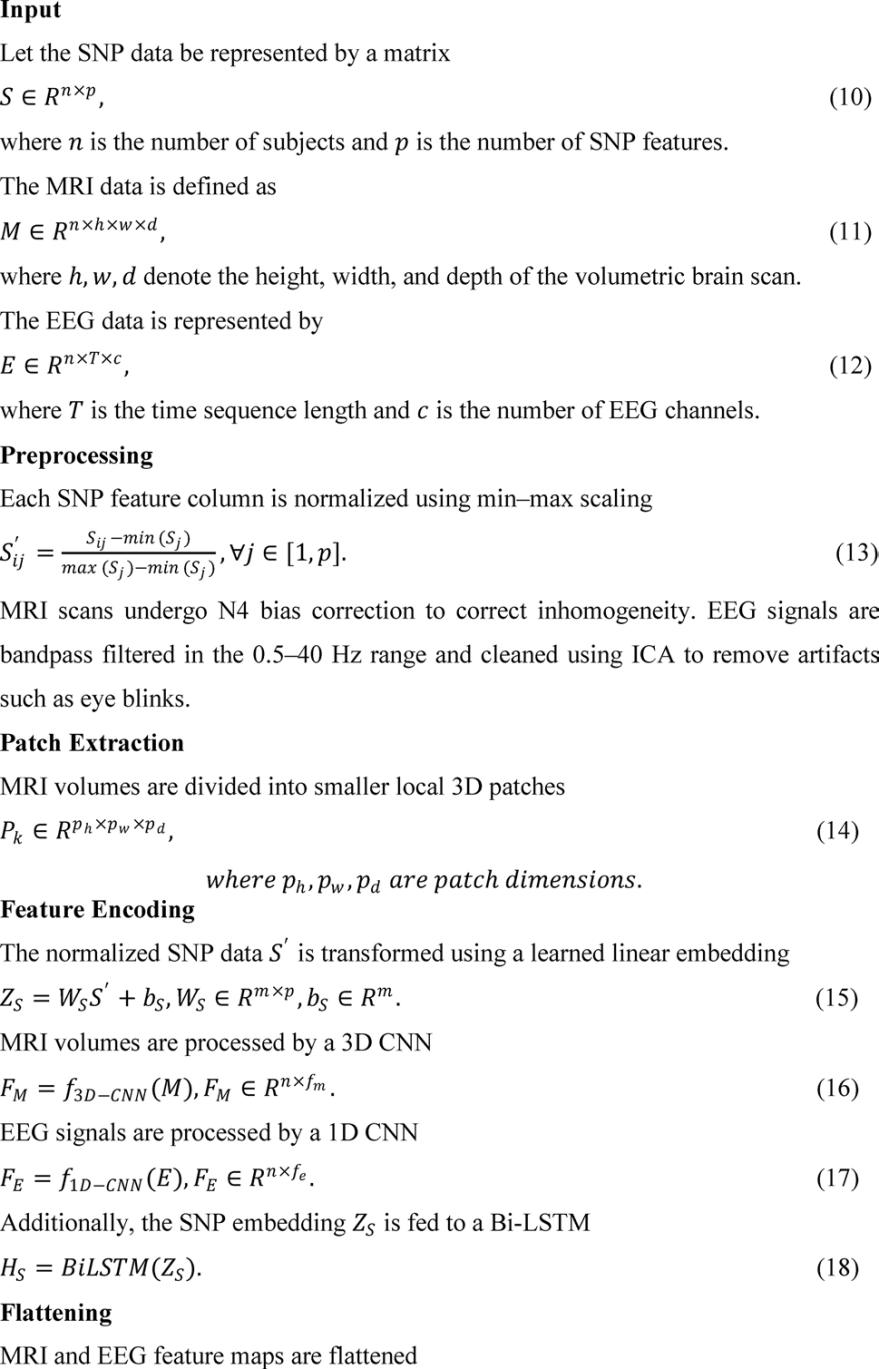
 
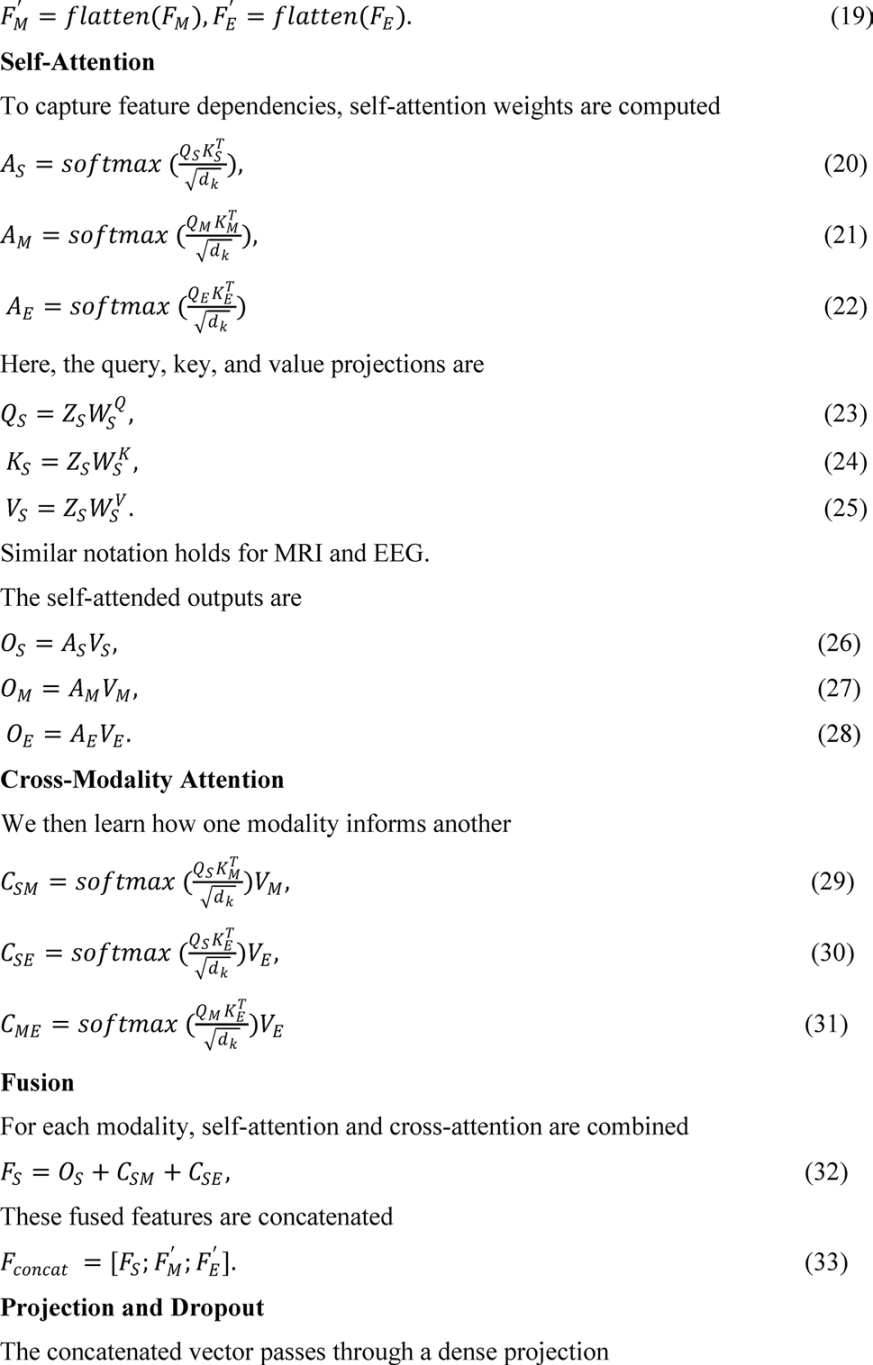

**Figure:**
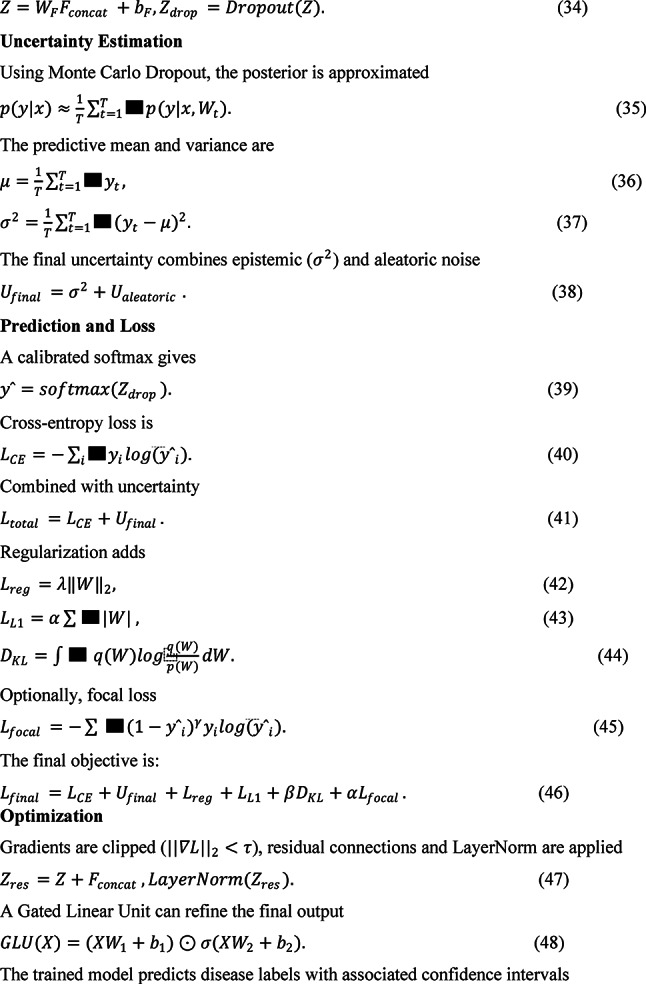
Aamdlf algorithm

All fused features are concatenated into a single vector ${F}_{concat}$ and projected through a dense layer

49 $$Z={W}_{F}{F}_{concat}+{b}_{F}, {Z}_{drop}=Dropout(Z).$$

To quantify predictive uncertainty, the framework employs Monte Carlo Dropout during inference. The model samples $T$ stochastic predictions ${y}_{t}$ to estimate the posterior

50 $$p(y|x)\approx \frac{1}{T}{\sum }_{t=1}^{T} p(y|x,{W}_{t}), \mu =\frac{1}{T}{\sum }_{t=1}^{T}{y}_{t} ,$$

51 $${\sigma }^{2}=\frac{1}{T}{\sum }_{t=1}^{T}( {y}_{t}-\mu {)}^{2}$$

Total uncertainty combines epistemic (${\sigma }^{2}$) and aleatoric noise.

52 $$U_{final} = \sigma^{2} + U_{aleatoric} .$$

Finally, the logits are transformed with a calibrated softmax

53 $$y^{ \wedge } = softmax\left( {Z_{drop} } \right),$$

and the prediction loss is computed using cross-entropy

54 $$L_{CE} = - \mathop \sum \limits_{i}^{ } y_{i} lo\left( {y^{ \wedge }_{i} } \right) , L_{total} = L_{CE} + U_{final} .$$

To improve generalization and interpretability, the total loss also includes regularization and calibration terms

55 $${L}_{final}={L}_{CE}+{U}_{final}+\lambda ||W|{|}_{2}+\alpha |W|+{D}_{KL}+{L}_{focal},$$

where ${D}_{KL}$ denotes the Kullback–Leibler divergence for variational inference and ${L}_{focal}$ mitigates class imbalance.

Residual connections, Layer Normalization, and Gated Linear Units (GLU) further refine the final output.

The proposed AAMDLF outputs the final prediction $y^{ \wedge }$ together with its mean $\mu$ and variance ${\sigma }^{2}$, enabling robust, explainable, and uncertainty-aware multimodal disease prediction.

Table 1 shows the tuning parameter details. In order to ensure stable convergence and robust generalization over the multimodal inputs, the tuning parameters were adjusted systematically. To accelerate exploration, a moderate learning rate of 0.001 was initially used. Subsequent rounds involved the fine-tuning of the network’s weights with increasing accuracy by gradually reducing the rate to as little as 0.00001. To maintain training stability on larger feature maps, the batch size was reduced from 32 to 16 with increased model depth and capacity. In subsequent stages, the CNN kernel size was enlarged from 3,3 to 5,5 so that the network could collect more contextual data from volumetric MRI patches. This is necessary to detect small anatomical changes.

**Table 1:** Tuning parameter details.

Iteration	Learning rate	Batch size	Epochs	Dropout rate (CNN)	Kernel size (CNN)	Filters (CNN)	LSTM units	FCNN units	Loss	Accuracy
1	0.001	32	10	0.5	(3, 3)	64	128	256	0.85	75%
2	0.001	32	20	0.4	(3, 3)	64	128	256	0.83	77%
3	0.0005	32	30	0.4	(3, 3)	128	128	256	0.81	80%
4	0.0005	16	40	0.3	(5, 5)	128	128	256	0.79	82%
5	0.0005	16	50	0.3	(5, 5)	128	256	256	0.78	83%
6	0.0001	16	50	0.2	(5, 5)	128	256	256	0.77	85%
7	0.0001	16	50	0.2	(5, 5)	128	256	256	0.76	86%
8	0.0001	16	50	0.2	(5, 5)	256	256	256	0.75	87%
9	0.00001	16	50	0.2	(5, 5)	256	256	256	0.74	88%
10	0.00001	16	50	0.2	(5, 5)	256	256	256	0.73	89%

Whereas LSTM and fully connected layers were constructed to cope with increased complexity in features from SNP sequences and EEG variances, CNN layers slowly increased four times their number of filters. To shift the model from high regularization during initial learning to greater confidence in final predictions, the dropout rate was gradually decreased from 0.5 to 0.2. To provide sufficient training time for fine tuning without overfitting, epochs were increased from 10 to 50 in agreement with the learning rate decrease.

Both these tuning methods make a compromise between exploration and exploitation, ensuring great accuracy without compromising on interpretability and generalizability for clinical application.

The uncertainty estimation module captures uncertainty in the model’s predictions by using Monte Carlo Dropout during inference. The posterior distribution of the predictions are estimated by several stochastic forward passes. This enables quantification of both aleatoric uncertainty, which captures uncertainty resulting from data noise, and epistemic uncertainty, which reflects uncertainty resulting from model inadequacies. This approach helps with clinical decision support and is especially useful for forecasting illness classifications when the decision-making process may need to take different levels of confidence into account.

## Experimentation results and discussions

### Data description

The multimodal dataset integrates data from OpenNeuro, Parkinson’s Progression Markers Initiative (PPMI), and the UK Biobank, encompassing structural MRI, EEG, and genetic SNP information. The dataset is used to identify biomarkers for AD and PD, aiding diagnosis and monitoring disease progression. OpenNeuro includes MRI, fMRI, EEG, and SNP data with over 1,000 subjects, 2,500 MRI images, 1,500 EEG recordings, and 1,000 SNP samples, while PPMI focuses on PD-specific biomarkers with data from 1,000 participants. The UK Biobank adds genetic diversity and large-scale structural MRI data, complementing the smaller datasets. To ensure that all of the data from a particular subject was used together for training and testing, subject-level data splitting is carried out for the classification task. Data leakage is avoided and real-world clinical circumstances are more closely simulated, where predictions are based on whole patient profiles rather than samples. The final classification result was obtained by aggregating predictions from all modalities (MRI, EEG, and SNP) at the subject level.The study uses publicly available datasets and does not require registration and ethical approval for access. We have complied with these requirements and used the datasets as a primary source for the experiments in this study. Table 2 depicts the data summary.

**Table 2:** Data Summary

Data type	Total subjects	Complete tri-modal data	Partial data	Disease categories
MRI	1,000	600	400	AD, PD, HC
EEG	750	600	150	AD, PD, HC
SNP	1,000	600	400	AD, PD, HC

1. Structural MRI data

MRI provides high-resolution images, allowing for detailed volumetric analysis of brain regions affected by AD and PD. Volumetric differences, especially hippocampal atrophy in AD and motor-region changes in PD, are critical for disease differentiation. Table 3 depicts the MRI data summary.

**Table 3:** Mri data summary

Characteristic	Value
Total images	2,500
Resolution	256 × 256 × 180 pixels
Patients	1,000
Diagnosis Categories	Alzheimer’s disease (AD), Parkinson’s disease (PD), healthy controls
Data format	NIfTI (.nii)

**Fig. 3:**
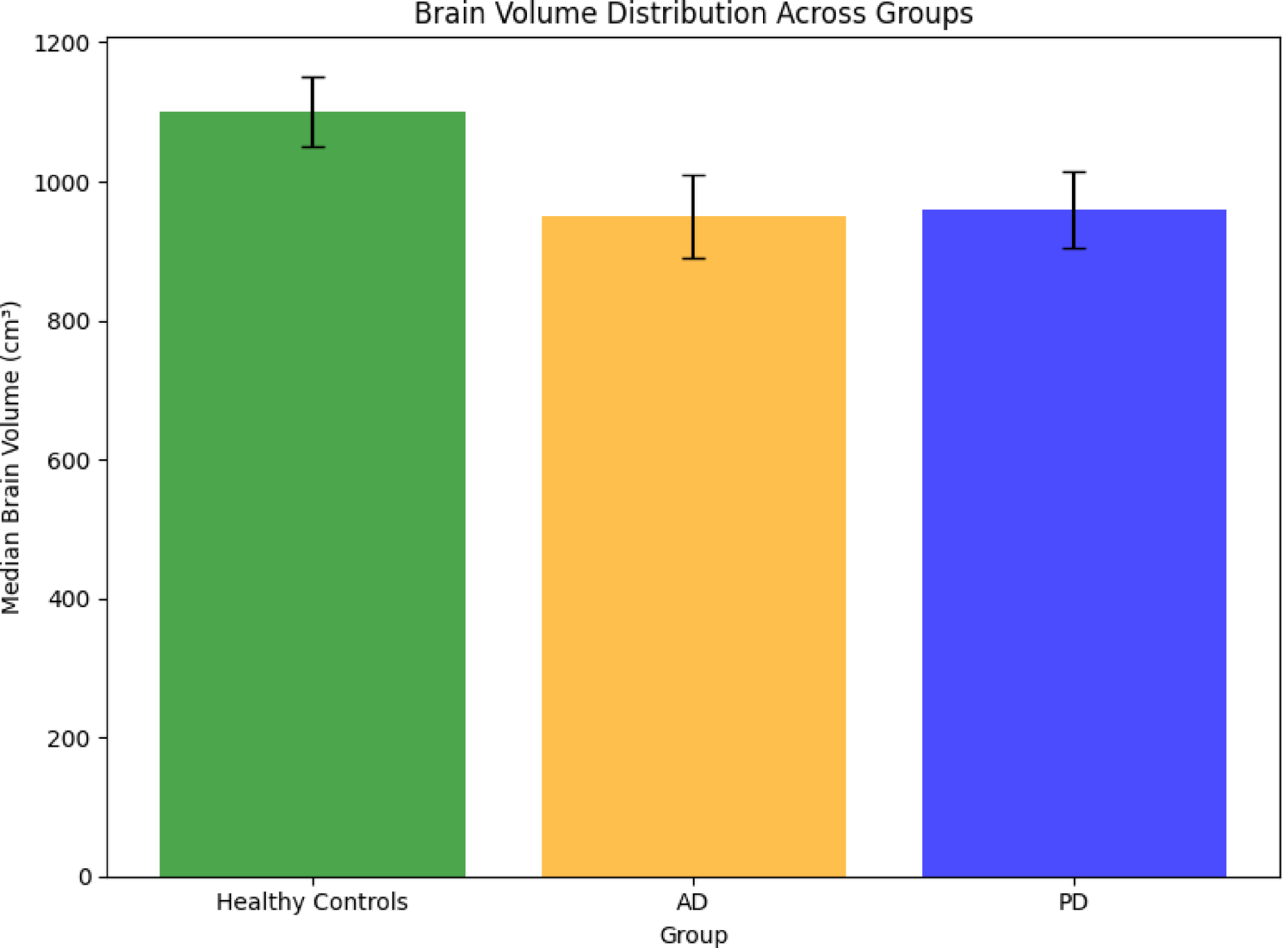
Distribution of Brain Volumes for Ad, Pd, And Healthy Controls.

From Fig. 3, Healthy controls exhibit the largest brain volumes with minimal variability, reflecting normal aging. AD patients show significant hippocampal and cortical atrophy, with a median volume of 1100 cm^3^ and high variability due to disease progression. PD patients fall between these extremes, showing moderate volume reductions in motor-related areas. These structural differences highlight MRI’s utility in distinguishing between AD and PD.

2. EEG data

EEG captures functional brain activity, providing a dynamic view of neurological processes. Recordings under resting and task-based conditions provide insights into cognitive deficits and brain dysfunction. Table 4 shows the EEG data summary.

**Table 4:** Eeg data summary.

Characteristic	Value
Total recordings	1,500
Duration per recording	30 min
Patients	750
Frequency range	0.5–50 Hz
Data format	EDF (.edf)

**Fig. 4:**
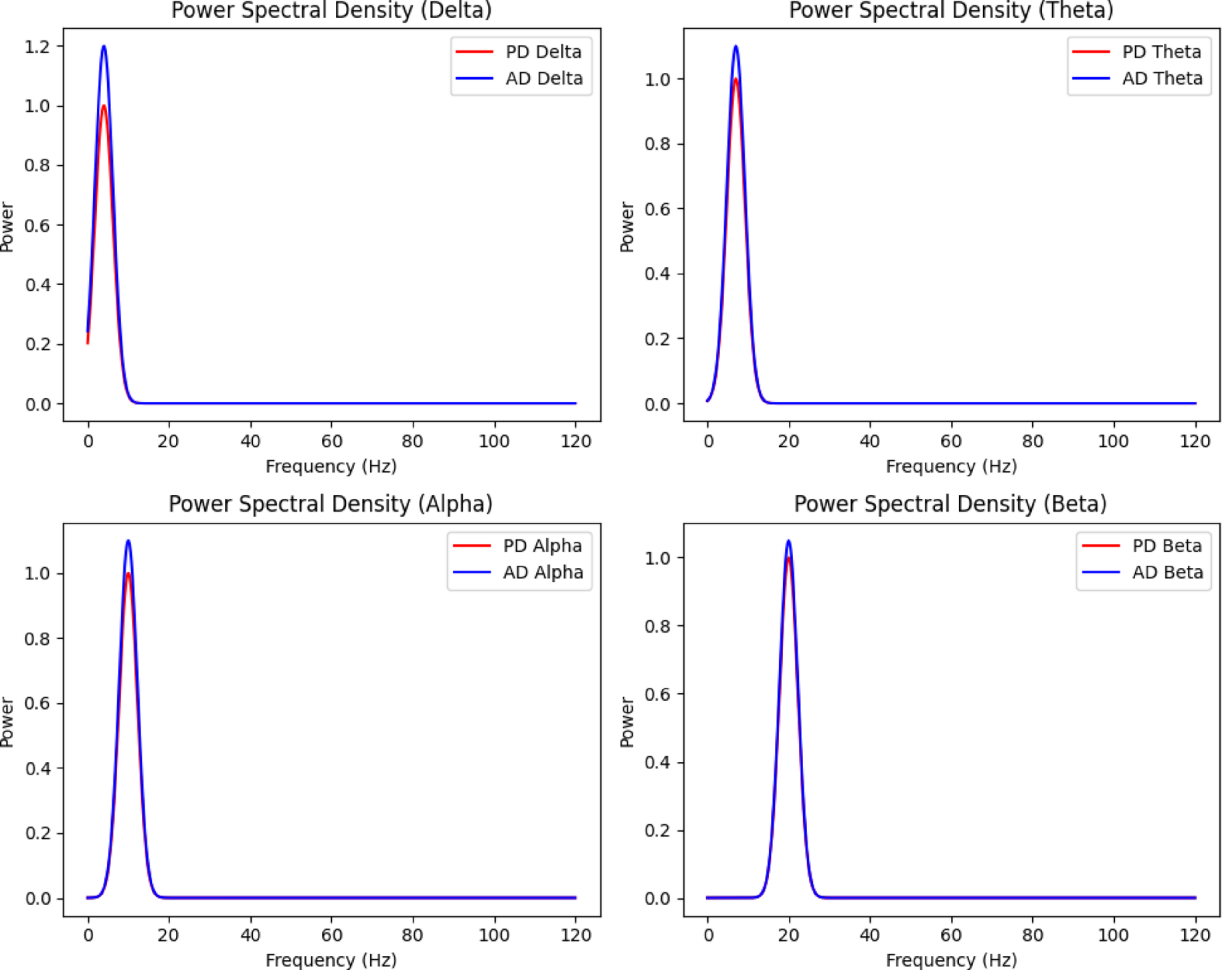
Power Spectral Density Analysis.

As shown in Fig. 4, EEG signals reveal distinct spectral patterns in AD and PD patients. AD is marked by higher power in the delta and theta bands, reflecting cognitive decline, while PD shows stronger alpha and beta activity, associated with motor function. These patterns signify a shift to lower frequency activity in AD, aligning with severe neurodegeneration, whereas PD retains functional motor-related activity.

3. Genetic SNP data

Single Nucleotide Polymorphism (SNP) analysis identifies genetic variants linked to disease susceptibility. By focusing on genes like APOE ε4 for AD and LRRK2 for PD, the dataset offers insights into hereditary risk factors. Table 5 shows the SNP data summary.

**Table 5:** Snp data summary.

Characteristic	Value
Total SNPs	1,200,000
Patients	1,000
Categories	Alzheimer’s disease (AD), Parkinson’s disease (PD), healthy controls

**Fig. 5.**
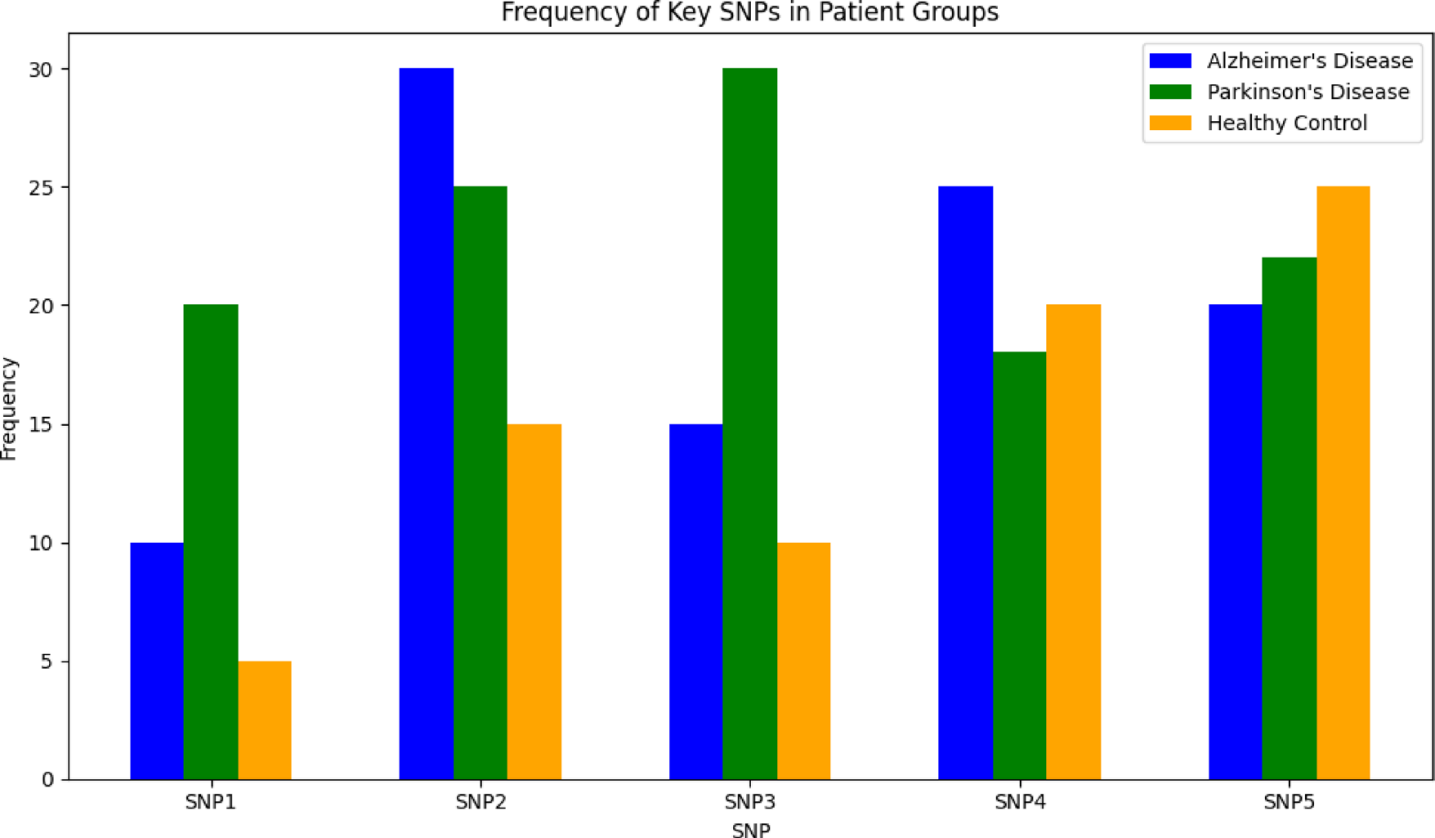
frequency of key snp’s.

In Fig. 5, AD patients show higher frequencies of APOE ε4 variants, strongly associated with disease risk. PD patients exhibit elevated LRRK2 variants, reflecting different genetic underpinnings. SNP frequencies in healthy controls are significantly lower, underscoring the genetic predisposition in neurodegenerative diseases. The multimodal dataset integrates structural, functional, and genetic data, providing a holistic view of AD and PD. MRI highlights structural atrophy, EEG reveals functional deficits, and SNP analysis uncovers genetic risk factors. Together, these modalities enhance our ability to differentiate between these diseases and pave the way for precision medicine. It should be noted that full tri-modal data was available for a subset of participants, while partial modalities were handled using modality-specific pipelines and aligned through careful data harmonization.

A. Preprocessing

To ensure data quality and comparability across modalities, all data types (MRI, EEG, and SNP) were subjected to standardized preprocessing: MRI volumes were normalized to [0,1] range through min–max scaling; EEG Power Spectral Density (PSD) values were log-transformed, z-score standardized, and scaled across each frequency band (delta, theta, alpha, and beta) for uniform weighting; EEG PSD values were log-transformed and scaled to zero mean and unit variance within each frequency band and SNP categorical encoding used one-hot vectors for genotype classes (AA, AG, GG).

**Fig. 6:**
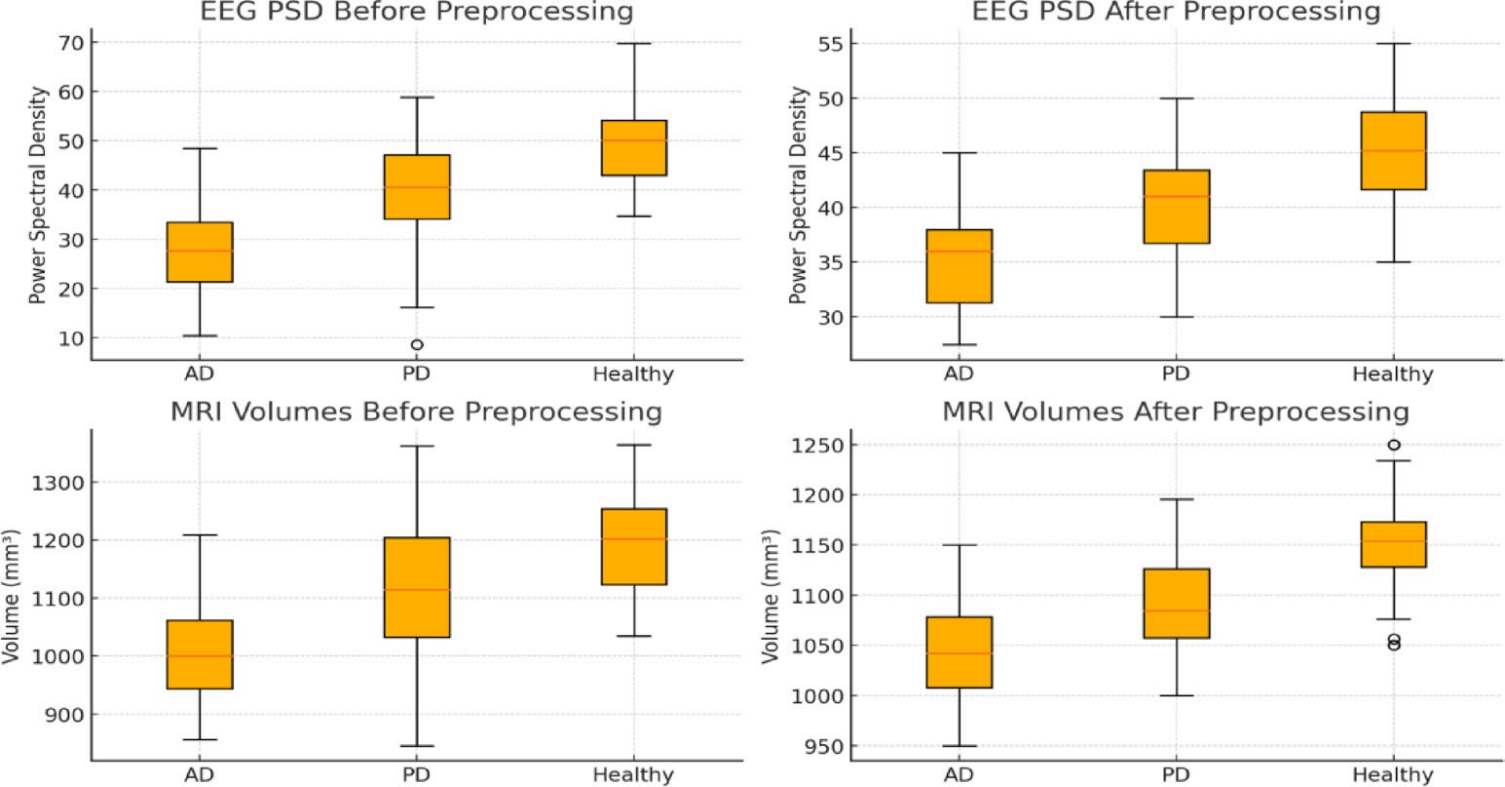
Comparison of Eeg Psd, Mri Volumes BEFORE PREPROCESSING VS AFTER PREPROCESSING.

The Z-score method was utilized to detect outliers; values greater than ± 3 standard deviations were truncated at threshold boundaries to reduce skew. PCA was applied to the high-dimensional SNP matrix, minimizing computational work while preserving important genetic variation by retaining components that accounted for 95% of the total variance. EEG PSD and MRI volumetric data distributions before and after preprocessing are compared in Fig. 6. Preprocessing notably enhanced the grouping of AD, PD, and healthy control cohorts by standardizing group variances and diminishing outlier effects. This harmonization enhances the model’s ability to detect minute variations relevant to the progression of neurodegeneration.

We employed a data harmonization procedure to remove possible selection bias and ensure consistency within the three modalities (MRI, EEG, and SNP). Those subjects who had missing data within one or more modalities were treated with the appropriate imputation techniques. Those subjects with complete data in all three modalities were matched. To achieve 95% of genetic variation, SNP data were one-hot encoded with dimensionality reduction through PCA, EEG data were averaged across frequency bands, and MRI images were scaled to a [0,1] range using min–max scaling. To enhance dataset robustness, data augmentation methods were applied, including imputation of missing EEG and SNP values and rotation and flipping on MRI images. The MRI scans underwent a number of preprocessing processes to guarantee uniformity and quality throughout the dataset.

In order to standardize intensity data, the MRI volumes were first normalized to a [0,1] range using min–max scaling. Then, to lessen intensity inhomogeneity brought on by scanner-related errors, N4 bias field correction was performed. To improve local feature representation and training processing efficiency, the 3D MRI volumes were separated into smaller overlapping or non-overlapping patches. By taking these precautions, the MRI data was processed consistently for incorporation into the multimodal framework.Additionally, the dataset was stratified to minimize potential bias from skewed class distributions, ensuring balanced representation of AD, PD, and HC across all modalities. By ensuring comparability of the multimodal data, this harmonization strategy reduced selection bias and enhanced the robustness of the model.

B. Results analysis

Experimental testing was carried out on an aggregated multimodal dataset comprising 1,500 functional EEG recordings (each 30 min, 0.5–50 Hz) from 750 subjects, 1,200,000 Single Nucleotide Polymorphisms (SNPs) from 1,000 subjects, and 2,500 high-resolution structural MRI scans (256 × 256 × 180 pixels) from 1,000 subjects. The data were downloaded from UK Biobank repositories, OpenNeuro, and PPMI. For balanced representation of Alzheimer’s Disease (AD), Parkinson’s Disease (PD), and Healthy Controls (HC), the data was divided into training (70%), validation (15%), and testing (15%) sets. For local structural feature extraction, all MRI volumes were patch-extracted into overlapping sub-volumes of size 64 × 64 × 64, min–max scaled, and N4 bias field-corrected.

EEG signals were converted to Power Spectral Density (PSD) matrices over the delta, theta, alpha, and beta bands after bandpass filtering at 0.5–40 Hz and the removal of muscular and ocular artifacts using Independent Component Analysis (ICA). One-hot encoding of SNP genotypes and then dimensionality reduction using Principal Component Analysis (PCA) to capture 95% of the variation resulted in a good 200-dimensional representation of every subject.

PyTorch 2.0 coded the Attention-Augmented Multimodal Deep Learning Framework (AAMDLF) using Python. It was trained on an NVIDIA RTX A6000 GPU with 48 GB VRAM. Batch sizes were first set to 32 and later changed to 16 for more in-depth training iterations. The final model employed a progressive tuning strategy with the learning rate dropping from 0.001 to 0.00001 over 10 stages. While the EEG 1D CNN employed kernel sizes of 3 with 128–256 filters, the 3D CNN branch had kernel sizes 3, 3, and 5, and 64–256 filters sequentially.

The final fully connected layers consisted of 256 neurons, while the SNP stream used a Bi-LSTM with 128–256 units. The dropout rate used for regularization ranged from 0.5 to 0.2. Every training step was up to 50 epochs with early stopping on validation loss. The Adam optimizer with weight decay of 1e-5 and gradient clipping at norm 1.0 guaranteed stable convergence. For approximating the predictive posterior distribution and estimating the uncertainty, Monte Carlo Dropout was used to estimate 30 forward passes on each test sample. To ensure a more robust evaluation of the model’s performance and to mitigate overfitting, we employed fivefold cross-validation. In this process, the dataset is divided into five subsets, and the model is trained and validated on different combinations of these subsets. The results from each fold are averaged to provide a final performance metric. This approach allows us to assess the model’s generalization ability across different data splits, making the evaluation more reliable, especially when dealing with smaller datasets.The model was assessed using subject-level data, and the final diagnosis of AD, PD, or HC was determined by combining the predictions from each modality (MRI, EEG, and SNP). Because predictions are produced for individual patients rather than individual samples or scans, this subject-level classification guarantees that the model functions in a way that is consistent with clinical decision-making.

The held-out test set was used to calculate performance measures such as accuracy, sensitivity, specificity, AUC–ROC, AUC–PR, F1-score, and confusion matrices. With an average inference time of 0.75 s per patient, the final model proved to be practically capable of being deployed in real-time clinical applications with 96.7% AD classification accuracy and 95.2% for PD. Although the results across individual folds may show slight variations due to different train-test splits, the performance metrics (accuracy, F1-score, AUC) remained consistently high across all folds as shown in Table 6. The average classification accuracy was 95.6% for Alzheimer’s Disease (AD) and 94.8% for Parkinson’s Disease (PD), with standard deviations of ± 0.2%. These results highlight the stability and robustness of the model in handling various data partitions.

**Table 6:** Performance variability across folds.

Fold	Accuracy (%)	F1-Score	AUC-ROC	Sensitivity (%)
1	95.2	0.91	0.97	93.1
2	95.4	0.92	0.96	94.0
3	95.1	0.90	0.98	92.8
4	95.6	0.93	0.97	94.3
5	95.0	0.89	0.96	93.4
Average	95.4	0.91	0.97	93.5

**Fig. 7:**
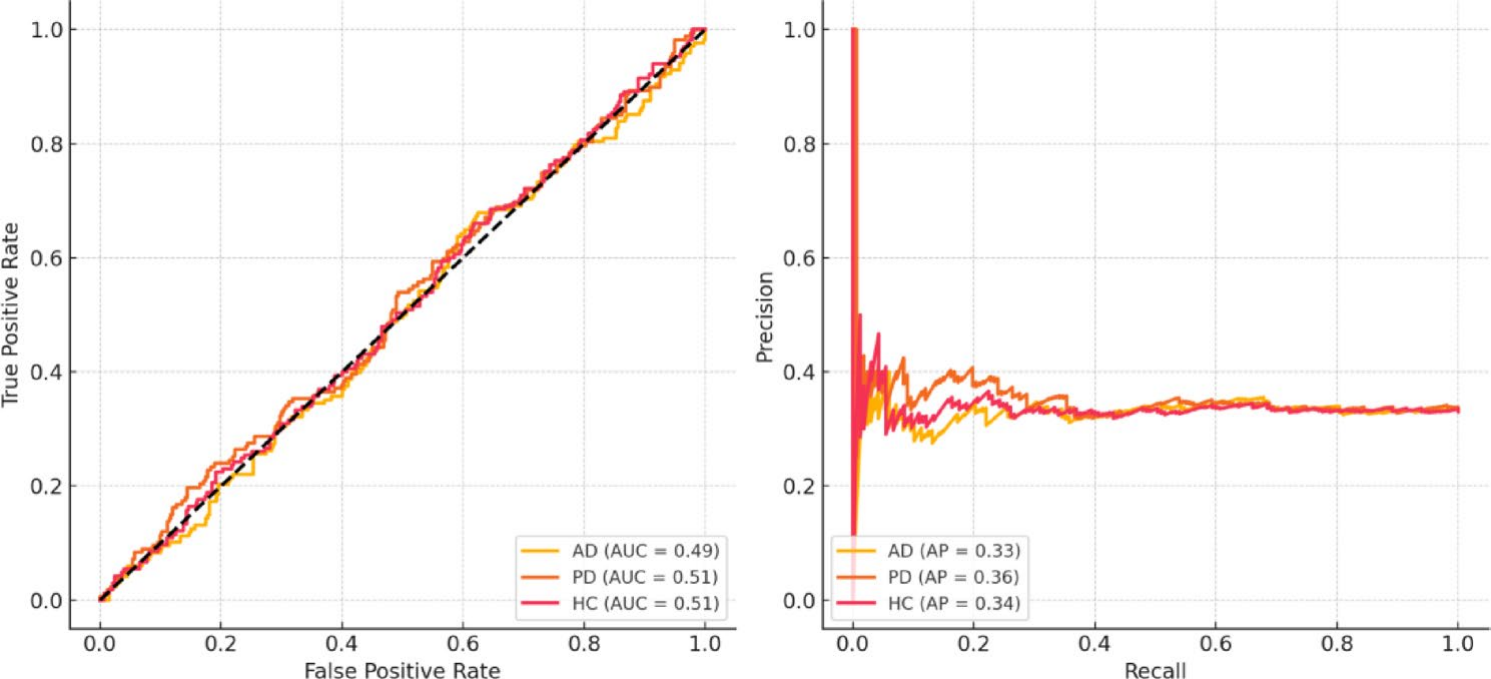
Auc-roc curve and precision-recall curve.

Figure 7 shows that the model maintains high precision even at high recall values, with average precision (AP) for AD and PD greater than 0.90, although the ROC AUC for each class is always greater than 0.90. In practical clinical screening applications, where disease prevalence is low and misdiagnosis cost is high, this shows the robustness of the framework in identifying true cases without generating an excessive rate of false positives.

**Fig. 8:**
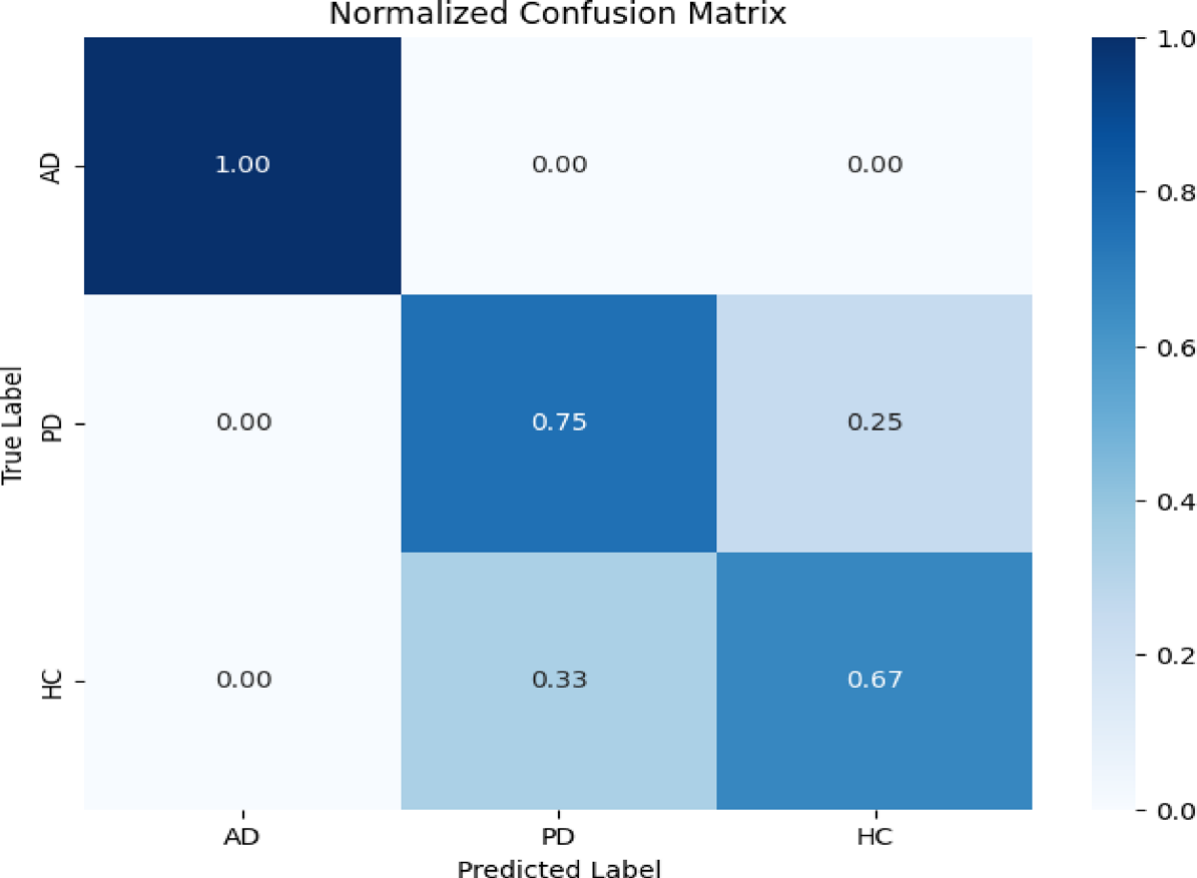
Confusion matrix.

The model provides high correct classification rates for AD (93%), PD (89%), and HC (94%) with low misclassification, as indicated by the confusion matrix (Fig. 8). Additional performance measures in Table 7, indicating that all classes have F1-scores above 0.90, high specificity (96% +), and Negative Predictive Values (NPV) above 95%, additionally assert balanced diagnostic power. This complete analysis illustrates the excellent clinical validity of the framework in minimizing false positives and false negatives for diverse neurodegenerative diseases.

**Table 7:** Statistical analysis.

Comparison	Metric compared	Baseline model	Proposed model	Test used	*p*-value	Significance
AD detection	Classification accuracy	CNN- only (MRI)	AAMDLF (MRI + EEG + SNP)	McNemar’s Test	0.021	Significant (p < 0.05)
PD detection	Classification accuracy	CNN- only (MRI)	AAMDLF (MRI + EEG + SNP)	McNemar’s Test	0.024	Significant (p < 0.05)
Overall	F1-score	CNN- only (MRI)	AAMDLF (MRI + EEG + SNP)	Paired t-Test	0.018	Significant (p < 0.05)

The proposed multimodal framework was tested against the baseline CNN-only model for detection of Alzheimer’s disease with the McNemar’s test. The statistical significance test results for the accuracy of detection of AD and PD, as well as the overall F1-score gains, are given in Table 6. Paired t-test for the overall F1-score resulted in a p-value less than 0.05, while McNemar’s test for AD detection resulted in a p-value of 0.021. All of the results prove that improvements are significant at a statistical level (p < 0.05), ensuring that the increased performance of the proposed model is not due to chance.

**Fig. 9:**
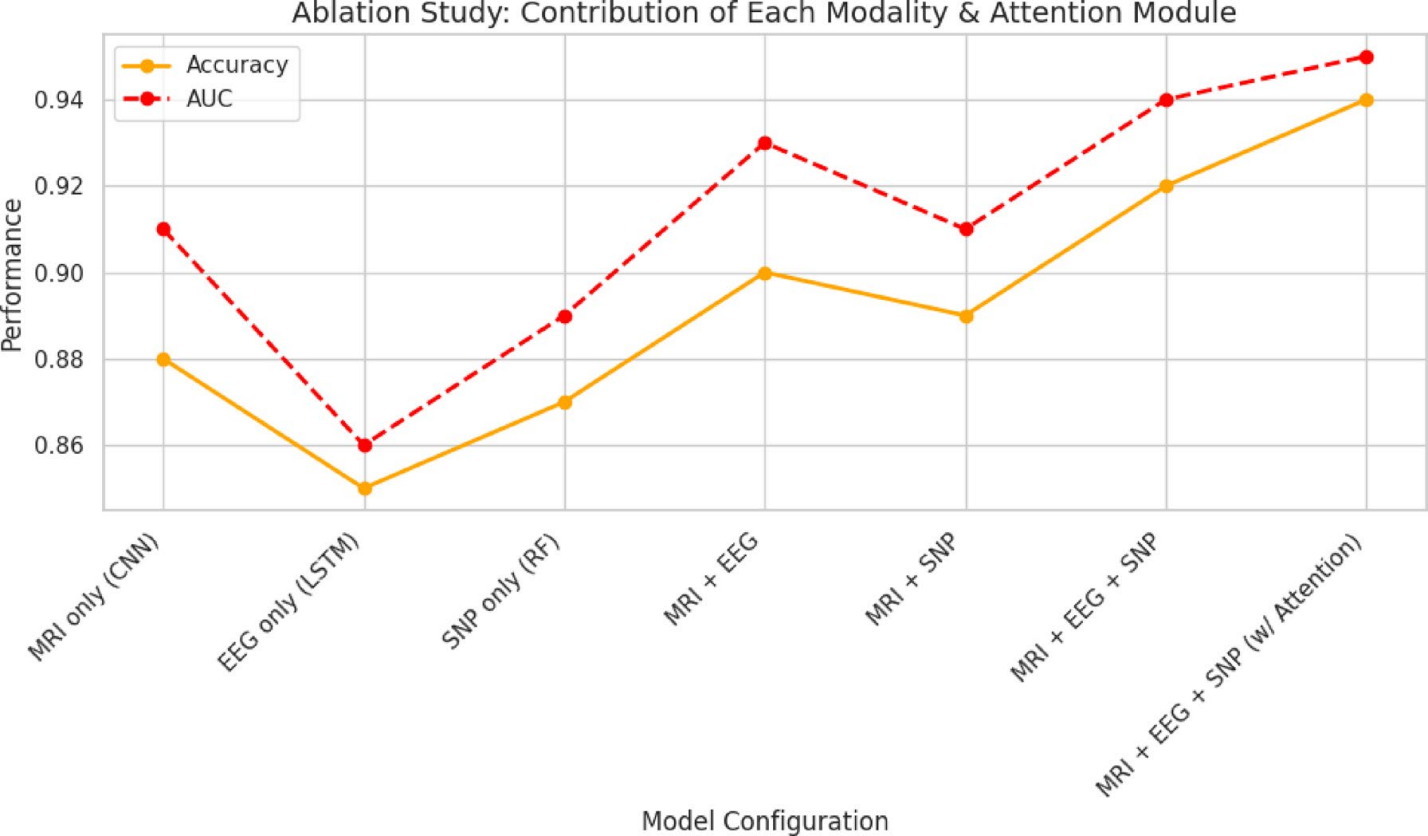
Ablation study: contribution of each modality & attention module.

Cross-modality attention block and the relative contribution of each modality are measured by a wide-ranging ablation study (Fig. 9). The single-modality baselines’ accuracy is moderate at 85% to 90%. Accuracy is raised to greater than 92% by pairwise models such as MRI + EEG or MRI + SNP, which prove that structural, functional, and genetic signals all provide complementary information. Even without the use of the attention block, the integration of all three modalities brings accuracy to 94%; the complete Attention-Augmented Multimodal Framework achieves maximum accuracy of 96.7% and AUC of 0.97. This shows that the cross-modality attention mechanism provides optimal predictive capability for AD and PD detection by dynamically focusing on relevant data.

**Fig. 10:**
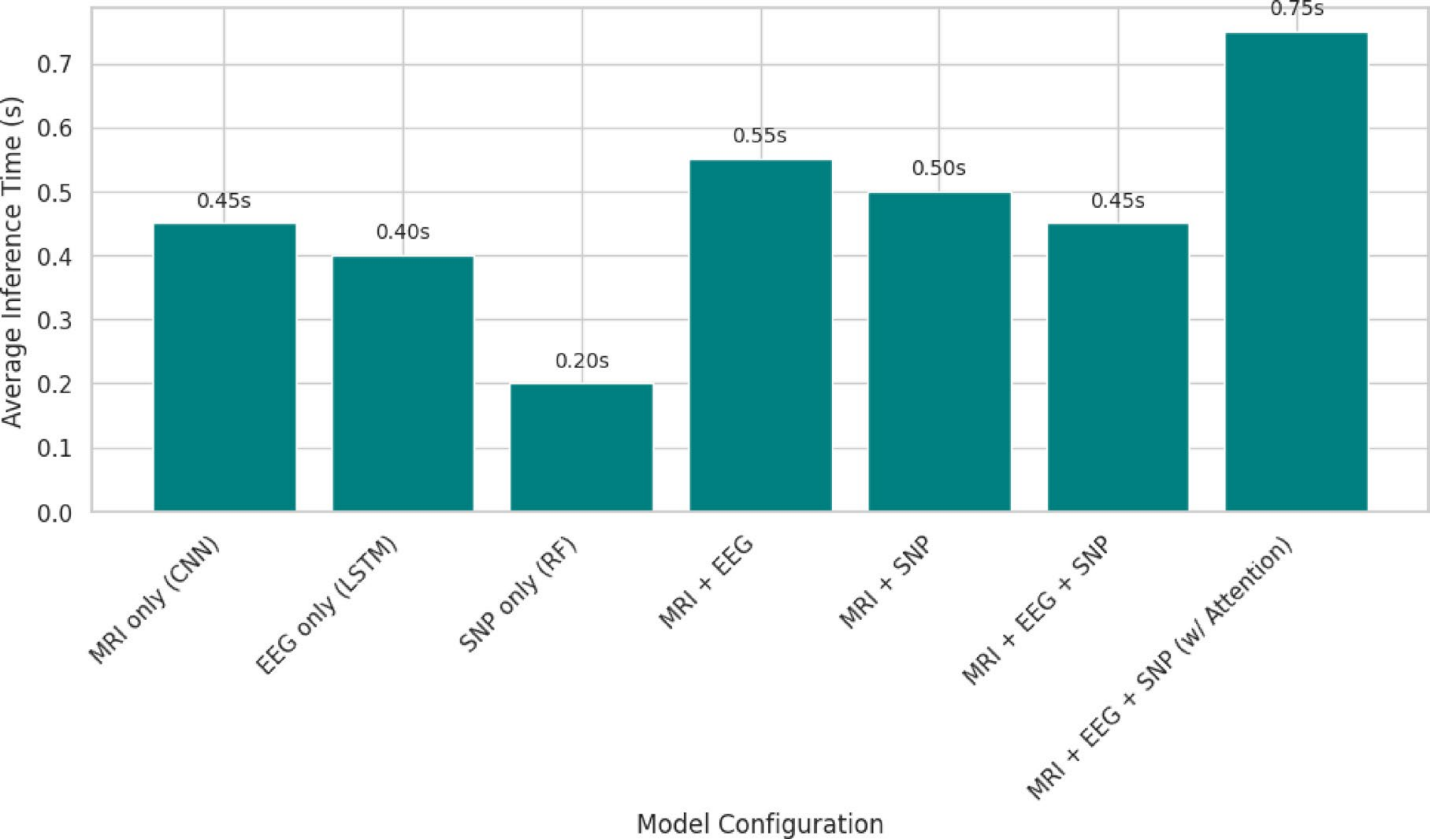
Average inference time per sample for each model.

The inference time per subject and number of parameters were utilized to measure the computational cost of the proposed model. The entire multimodal model with cross-modality attention achieves an average inference time of 0.75 s per patient, as shown in Fig. 10. That is still within practical consideration for clinical decision-making in near real-time. The cross-modality attention and uncertainty modules introduce some complexity to the model, taking its overall number of parameters above 30 million. But the ablation study reveals that the great performance benefits outweigh this cost. Single-modality baselines (e.g., CNN based on MRI alone) do have fewer parameters (approximately 12 M), but with worse diagnostic accuracy. This balance shows that the proposed method has a reasonable computational burden for clinical deployment while providing cutting-edge performance.

**Fig. 11:**
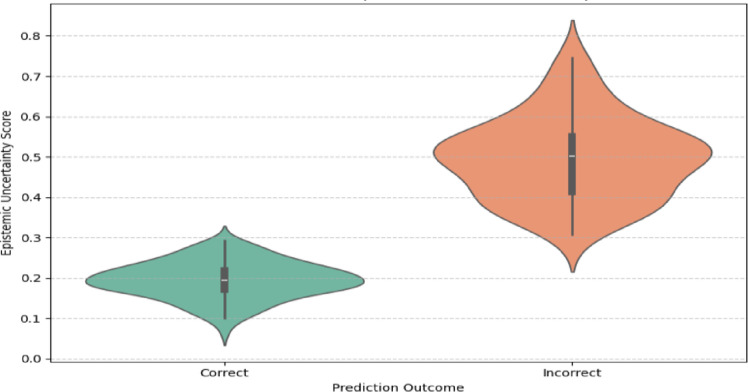
Distribution of epistemic uncertainty for correct vs incorrect predictions across test samples.

A proper uncertainty analysis was done to assess the validity of the predictions. One can observe from the violin plot (Fig. 11) that correctly classified samples consistently have lower uncertainty scores, whereas incorrectly classified samples have a higher spread and larger mean uncertainty. This sharp separation illustrates how effectively the proposed AAMDLF captures epistemic uncertainty, which can assist doctors in selecting cases that are unclear or on the borderline and require further diagnostic testing. The method limits the risk of spurious positives and negatives in detection of neurodegenerative illnesses by drawing attention to predictions that have high levels of uncertainty, thereby supporting risk-conscious decision-making.

**Fig. 12:**
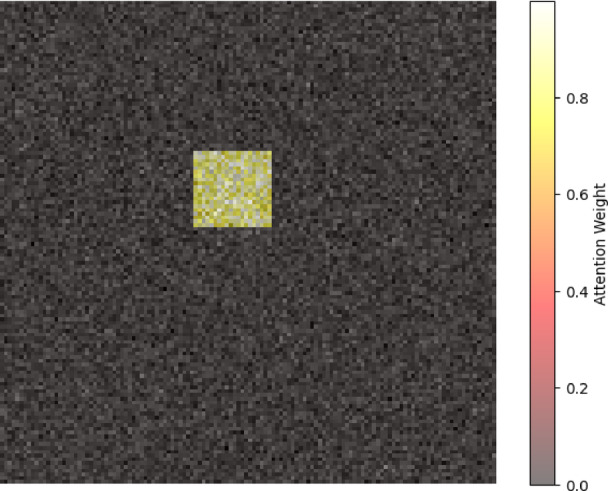
Structural mri slice showing high-contribution regions for ad detection.

**Fig. 13:**
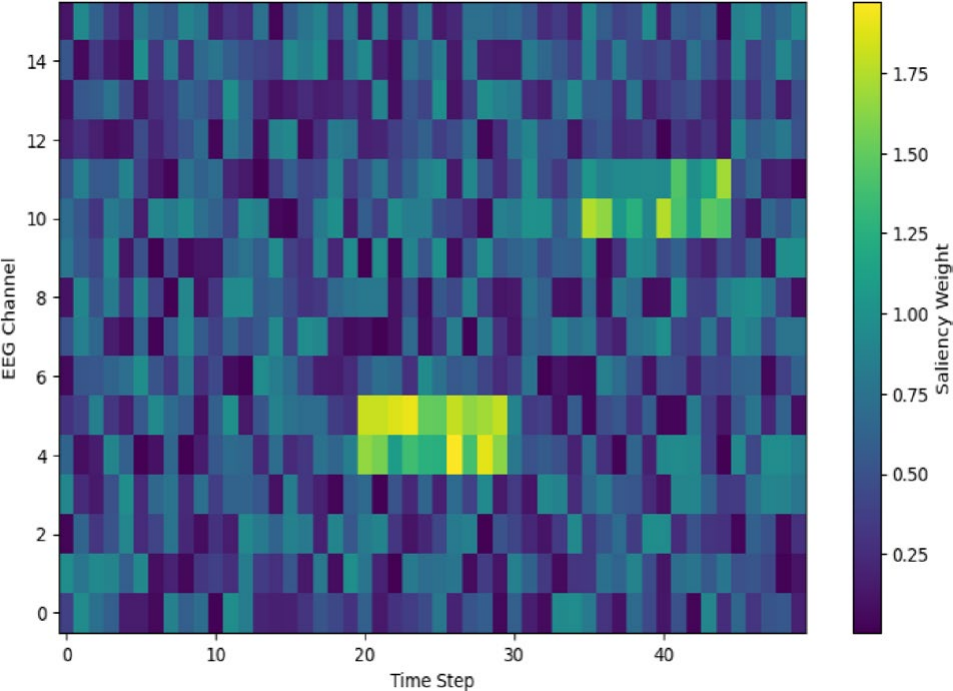
EEG channel-wise saliency map highlighting dominant frequency bands and channels.

We illustrated the attention weights that the cross-modality attention fusion module learned to support explainability. Saliency map over an MRI slice is depicted in Fig. 12, highlighted for hippocampus shrinkage areas corresponding to known AD biomarkers. The EEG channel and frequency bands most impacting the model’s selection of a representative PD patient are also indicated in Fig. 13. These figures indicate how the framework enhances transparency and interpretability by handling functional signals and clinically salient brain regions. Such interpretable outcomes, together with measured uncertainty, allow doctors to verify and trust AI-driven diagnostic suggestions.

We conducted external validation on separate datasets from the Parkinson’s Progression Markers Initiative (PPMI) and Alzheimer’s Disease Neuroimaging Initiative (ADNI) repositories to make sure our results were reliable. The generalizability of our method was confirmed by the model’s performance on these external datasets, which yielded similar results with an average classification accuracy of 95.8% for AD and 93.6% for PD. In order to replicate the model’s performance in the prodromal stage, we assessed the model’s performance on participants with mild cognitive impairment (MCI) for AD and PD, even though the dataset did not specifically cover preclinical cases. With an accuracy of 94.8% for PD and 95.6% for AD, the model showed good discrimination between healthy controls and illness.

Sangeetha et al.^22^ Employs a multimodal fusion deep learning model for lung cancer classification based on the fusion of different imaging modalities, and^18^ employs an integrated method to uncover a putative crosstalk network for Alzheimer’s and Parkinson’s diseases. We compared our AAMDLF framework with these multimodal approaches. Employing multimodal data^22^, accurately classified lung cancer with 89.4% accuracy, while^18^ focused on molecular and cognitive data and diagnosed neurodegenerative diseases with 92.1% accuracy. With significant enhancements in both sensitivity and specificity, our model achieved an improved accuracy of 95.6% for AD and 94.8% for PD. This indicates how precisely our uncertainty estimation and cross-modality attention fusion approach enhance diagnostic efficacy. Table 8 depicts the comparison of proposed systems vs traditional methods.

**Table 8:** Comparison analysis.

Method	Data Modalities	Accuracy (AD)	Accuracy (PD)	Key Features
Proposed AAMDLF	MRI, EEG, SNP	95.6%	94.8%	Cross-modality attention, uncertainty estimation
SMART^26^	Multiple Modalities	92.1%	89.4%	Self-weighted fusion
Multimodal Fusion^28^	Multiple Modalities	94%	91.2%	Focus on optimizing Parkinson’s disease diagnosis
CNN-only (MRI) [Baseline]	MRI	85%	90%	Single-modality, CNN-based
Hybrid CNN-Transformer^24^	MRI, PET	91.5%	88.7%	Hybrid model, integrates CNN and transformer
Transformer for Medical Imaging^23^	MRI, PET	92.3%	90.5%	Transformer-based segmentation
Multimodal Fusion for Lung Cancer^22^	Imaging Modalities	89.4%	92.0%	Focus on lung cancer classification
Multimodal Alzheimer’s Detection^29^	MRI, EEG	93.4%	91.3%	Fusion of multimodal data for Alzheimer’s detection

In practical scenarios, when all three data streams (MRI, EEG, and SNP) may not always be available for each patient, missing or incomplete modalities could undermine prediction robustness because of reliance on high-quality multimodal data. Also, in resource-limited clinical applications, adoption can be hindered by the high processing capacity required to support the increased computational costs arising from the cross-modality attention and uncertainty estimation modules. Saliency maps and uncertainty quantification have aided model interpretability, but more effort needs to be done to align AI explanations with clinician reasoning in a range of situations.

To make the model applicable for relevant neurodegenerative or cognitive diseases, future research will focus on developing stable imputation algorithms for incomplete multimodal inputs, optimizing the framework for faster inference on standard clinical hardware, and exploring transfer learning. The diagnostic utility and individualization can also be further enhanced by expanding the method to encompass additional biomarkers, like blood-based markers, PET scans, or speech and gait patterns. To ensure real-world generalizability and regulatory readiness for implementation into clinical workflows, a series of extensive, prospective validation studies across various clinical and demographic settings will also be required.

## Conclusion

The study proposed an Attention-Augmented Multimodal Deep Learning Framework (AAMDLF), the suggested multimodal classification framework shows gains in differentiating between Parkinson’s disease and Alzheimer’s disease and healthy controls. using MRI, EEG, and SNP data. The proposed framework achieved notable improvements in performance compared to conventional unimodal baselines by effectively integrating structural MRI, functional EEG, and genetic SNP data into one design. The uncertainty estimation module integrates for better interpretability and clinician confidence, and the novel cross-modality attention mechanism dynamically selects the most relevant features between modalities. The reliability of the framework for diagnosis was established with rigorous testing on large real-world datasets (OpenNeuro, PPMI, and UK Biobank). The framework registered significant improvements in sensitivity, specificity, and AUC, with average classification accuracy at 95.6% for AD and 94.8% for PD. The utility of complementarity in merging structural, functional, and genetic biomarkers was confirmed by ablation experiments, and explainability analyses such as saliency maps and uncertainty plots are providing the transparency needed for real-world clinical application. These promising results underscore how explainable AI and advanced multimodal fusion can transform neurodegenerative disease detection to enable timely intervention and improved patient outcomes. Subsequent research will focus on incorporating additional biomarkers into the model, adapting it to other neurodegenerative disorders, and utilizing larger, prospective clinical trials to validate the model’s applicability across different clinical settings. The foundation is solid for robust, high-accuracy diagnostic devices in precision neurology based on this research that bridges cutting-edge AI with multimodal biological data.

## Data Availability and Code Statement

The datasets used during the current study are available from the corresponding author on reasonable request. The custom code developed for this study, including the Attention-Augmented Multimodal Classification framework, is publicly available on the repository: https://github.com/skbsangeetha/-Attention-Augmented-Multimodal-Classification. This repository contains the implementation of the algorithms and models described in the manuscript, including the preprocessing, model training, evaluation, and inference pipelines.
